# Current Status of Maternal Gestational Weight Gain and Obstetric Outcomes in Japan

**DOI:** 10.7759/cureus.48988

**Published:** 2023-11-18

**Authors:** Shunji Suzuki

**Affiliations:** 1 Obstetrics and Gynecology, Nippon Medical School, Tokyo, JPN

**Keywords:** low-birth-weight infants, japan, body mass index, pregnant women, gestational weight gain

## Abstract

Introduction

In 2021, the Japanese Ministry of Health, Labour and Welfare (JMHLW) revised the standard optimal gestational weight gain (GWG) to reduce the incidence of low-birth-weight infants (LBWI) in Japan. In this study, we examined whether maternal GWG increased and LBWI decreased after the revision.

Materials and methods

We reviewed the obstetric records of singleton pregnant Japanese women who delivered at our institute at ≥37 weeks’ gestation in 2020 (before revision) and 2022 (after revision).

Results

The maternal GWG was significantly increased after the revision of the JMHLW guideline; however, the expected decrease in the incidence of LBWI was not achieved.

Conclusion

The maternal GWG met the new criteria in the revised guidelines and did not appear to contribute to the reduced incidence of LBWI.

## Introduction

Poor maternal gestational weight gain (GWG) during pregnancy has been reported to be associated with a higher frequency of small-for-gestational-age infants [1-3]. Although Japan had been one of the few developed countries with an increased incidence of low-birth-weight infants (LBWI), the recommended GWG for Japanese women was smaller than those in Western countries until 2020 [4-6]. For example, in the Japanese Ministry of Health, Labour and Welfare (JMHLW) guideline, optimal ranges of GWG in underweight (BMI; kg/m^2^, BMI: <18.5) and normal women (BMI: 18.5-24.9) had been suggested as 9-12 kg and 7-12 kg, respectively [7]. In addition, in the Japan Society for the Study of Obesity (JASSO) guideline, optimal ranges of GWG in overweight (BMI: 25.0-29.9) and obese women (BMI ≥30) had been suggested as ≤7 kg and ≤5 kg, respectively [8].

Based on some recent reports highlighting the trend as a serious problem, in 2021 the JMHLW revised the optimal GWG standard [9-13]. With the revision, the optimal ranges of GWG in underweight, normal, overweight, and obese women were changed to 12-15 kg, 10-13 kg, 7-10 kg, and ≤5 kg, respectively [10,14]. However, it is still unclear whether the number of LBWI has decreased since the revision. At our institute, we have distributed a leaflet with graphs of optimal GWG standards for each period to all pregnant women during their first prenatal health consultation. Therefore, in the current study, we examined whether maternal GWG increased and LBWI decreased after the revision.

## Materials and methods

The protocol for this study was approved by the Ethics Committee of the Japanese Red Cross Katsushika Maternity Hospital　(K2023-17). Informed consent concerning the analysis of anonymously processed information from a retrospective database was obtained from all subjects.

We reviewed the obstetric records of all singleton pregnant Japanese women who delivered at our institute (one of the major perinatal centers in Tokyo, Japan) at ≥37 weeks’ gestation in 2020 (before revision) and 2022 (after revision).

As characteristics of the pregnant women, we examined maternal age at delivery, primiparous rate, maternal height, body weight, and BMI at pre-pregnancy. The pregnant women were categorized by their physique at pre-pregnancy. Their average GWG was calculated for each physique and compared to the GWGs and the GWG-related obstetric outcomes between the two groups in 2020 and 2022. The main obstetric outcomes were hypertensive disorders, gestational diabetes mellitus (GDM), the rate of cesarean delivery, the incidence of postpartum hemorrhage ≥1,000 mL, neonatal birth weight, the incidence of LBWI, and the incidence of neonatal asphyxia. Gestational age was calculated using the ultrasonographic findings at nine to 11 weeks gestation. GDM was diagnosed when at least one of the following was found: fasting blood glucose level of ≥92 mg/dL, blood glucose level at one hour of ≥180 mg/dL, and blood glucose level at two hours ≥153 mg/dL by 75 g oral glucose tolerance test. Low birth weight was defined as a neonatal birth weight of <2,500 g. Neonatal asphyxia was defined as a neonatal Apgar score at one or five minutes of <7.

Data were expressed as mean ± standard deviation (SD) or number (percentages). Cases and controls were compared by means of Student’s t-test for continuous variables, and the χ^2^ or Fisher’s exact test for categorical variables. Odds ratios (ORs) and 95% confidence intervals (CIs) were calculated. Differences with p < 0.05 were considered significant.

## Results

We managed 1,487 and 1,492 singleton pregnant Japanese women at our institute at ≥22 weeks gestation in 2020 and 2022, respectively. Of these, 1,330 (89.4%) and 1,364 (91.4%) were delivered at ≥37 weeks gestation in 2020 and 2022, respectively. Therefore, there was no significant difference in the incidence of preterm delivery between the two periods (p = 0.07).

Table 1 shows the clinical characteristics of singleton pregnant Japanese women who delivered at our institute at ≥37 weeks gestation in 2020 and 2022. The average maternal age in 2022 was higher than that in 2020 (p < 0.02); however, there were no significant differences in other variables between the two periods.

**Table 1:** Clinical characteristics of singleton pregnant Japanese women who delivered at our institute at ≥37 weeks gestation in 2020 and 2022 Data are presented as number (percentage) or mean ± SD. SD, standard deviation

Study period (year)	2020	2022	P-value
Total number	1,330	1,364	
Primiparous women	652 (49.0)	670 (49.1)	0.96
Maternal age (y)	31.9 ± 5.4	32.1 ± 5.2	0.02
Gestational age at delivery (w)	39.3 ± 2.0	39.2 ± 1.9	0.06
Maternal height (cm)	158.3 ± 5.5	158.4 ± 5.8	0.27
Maternal weight at pre-pregnancy (kg)	54.2 ± 8.0	54.3 ± 7.9	0.36

Table 2 shows the prevalence of the mothers’ physique at pre-pregnancy in singleton pregnant Japanese women in 2020 and 2022. There were no significant differences in these variables between the two periods (p = 0.93).

**Table 2:** The prevalence of the mothers’ physique at pre-pregnancy in singleton pregnant Japanese women who delivered at our institute at ≥37 weeks gestation in 2020 and 2022 Data are presented as number (percentage).

Study period (year)	2020	2022
Total number	1,330	1,364
Underweight	125 (9.4)	130 (9.5)
Normal	1,038 (78.0)	1,067 (78.2)
Overweight	137 (10.3)	138 (10.1)
Obese	30 (2.3)	29 (2.1)

Table 3 shows the average GWG of the mothers’ physique at pre-pregnancy. The average maternal weight at delivery in 2022 was heavier than that in 2020 (65.5 ± 9.6 vs. 65.0 ± 9.1 kg, p < 0.01). As shown in Table 3, the GWGs in 2022 were higher than those in 2020 in the underweight, normal, and overweight women (p < 0.01).

**Table 3:** The average GWG in singleton pregnant Japanese women who delivered at our institute at ≥37 weeks gestation in 2020 and 2022 of the mothers’ physique at pre-pregnancy Data are presented as mean ± SD. GWG, gestational weight gain; SD, standard deviation

Study period (year)	2020	2022	P-value
Average (kg)	10.8 ± 2.4	11.2 ± 2.8	<0.01
Underweight (kg)	12.0 ± 2.6	12.8 ± 2.4	<0.01
Normal (kg)	10.7 ± 2.5	11.1 ± 2.5	<0.01
Overweight (kg)	6.0 ± 3.0	8.2 ± 2.6	<0.01
Obese (kg)	2.0 ± 2.6	2.5 ± 2.1	0.22

Table 4 shows the obstetric outcomes including the incidence of LBWI. The rate of cesarean delivery in 2022 was higher than that in 2020. The average neonatal birth weight in 2022 was statistically lighter than that in 2020, and there was no significant difference in the incidence of LBWI. There were no significant differences in other variables between the two periods.

**Table 4:** The obstetric outcomes in singleton pregnant Japanese women who delivered at our institute at ≥37 weeks gestation in 2020 and 2022 Data are presented as number (percentage) or mean ± SD. LBWI, low-birth-weight infants; SD, standard deviation

Study period (year)	2020	2022	P-value
Total number	1,330	1,364	
Hypertensive disorders	90 (6.8)	97 (7.1)	0.76
Gestational diabetes	33 (2.5)	31 (2.3)	0.72
Cesarean delivery	452 (34.0)	531 (38.9)	<0.01
Neonatal birth weight			
Average (g)	3,041 ± 481	3,029 ± 458	0.03
LBWI	84 (6.3)	87 (6.4)	0.95
Neonatal asphyxia			
Total	18 (1.4)	16 (1.2)	0.67
LBWI only	2 (/84, 2.4)	2 (/87, 2.3)	0.97

## Discussion

In this study, the maternal GWG increased significantly after the revision of the JMHLW guideline met the new optimal GWG criteria for all groups. However, the expected decrease in the incidence of LBWI was not achieved. Although only one year has passed since the revision of the JMHLW guideline, the effects of nutritional guidance for Japanese mothers seem to be well recognized. However, the positive impact of the revised guidelines did not extend to the incidence of LBWI.

It has been suggested that one of the main reasons for the high incidence of LBWI in Japan is the desire of young women to be thin (low body weight) [15,16]. Young Japanese women equate thinness with beauty; hence, they eat unreasonably to get slim [17,18]. According to a Japanese government survey, one in five young Japanese women skipped breakfast, and their intake of protein, vegetables, and calcium was low [17,18]. The JMHLW guideline was revised with an emphasis on the necessity for the optimal GWG by pregnant women; however, the optimal GWG may not be necessarily associated with the ideal dietary patterns and/or nutritional balance [10,14]. The GWG will be just one of the effects of nutritional intake. In addition, various studies conducted in Japan have shown that there are regional differences in the optimal amount of GWG, and it was assumed to be due to differences in dietary habits [12,13]. Therefore, pregnant women should be advised about optimally balanced nutritional diets to help them achieve the ideal GWG.

In this study, the 2022 group had more older women compared with the 2020 group. In addition, the 2022 group had earlier gestational week at deliveries although the differences did not reach statistical significance. These have been reported to be associated with the increased risk of LBWI [19-22]. This may be one of the limitations explained later. If the sample size increases, these differences become significant, and with multivariate analysis, the incidence of LBWI may decrease after the revision of the guideline.

We understand that there are some limitations such as the small sample size of the study mentioned above. In this study, to examine the incidence of LBWI as a primary outcome, we excluded the cases of preterm delivery. We confirmed that there was no difference in the incidence of preterm delivery between the two groups; however, to examine the influence of GWG on obstetric outcomes in detail, the clinical parameters of preterm delivery may need to be further examined [22]. In Japan, there are regional differences in nutritional intake as mentioned above. We may also need to consider other factors related to LBWI such as smoking [3]. In addition, there are various types of obstetric institutes, with half of the deliveries managed in private clinics, and the pregnant women who give birth at perinatal centers often have some perinatal serious risk factors [23]. Therefore, the current results observed in our institute may not reflect the trends as a whole in Japan.

## Conclusions

In conclusion, the maternal GWG increased significantly after the revision of the JMHLW guideline, but the expected decrease in the incidence of LBWI was not achieved. However, the nutritional guidance for Japanese mothers appears to have a positive impact on GWG but not on the incidence of LBWI.
